# Hospital Meetings, &c.

**Published:** 1899-04-22

**Authors:** 


### HOSPITAL MEETINGS, &C.

### THE SAMARITAN FREE HOSPITAL.

The annual meeting of the governors of the Samaritan Free Hospital was held at the hospital on Tuesday, April 18th, 1899. Dr. Savage took the chair, and made a few remarks upon the report. He said that this was the fifty-second occasion of their annual meeting, and that with few exceptions he had found himself in the chair. Last year the hospital had been heavily in debt, but he was glad to see that it was now financially solvent. He noticed some stringent remarks in the report as to the state of the out-patient department. Every year something of the kind was said, and for his part he would be glad to have the subject shelved for a time, for until some leases fell in and gave them the possession of a site the governors could make no move to remedy the matter. He referred to the late Sir Spencer Wells and remarked that the obituary in the *Lancet* failed to do him justice. It drew attention to the methods of Sir Spencer, whereas it was the principle on which he worked that has created ovarian surgery. After the re-election of the committee of management, Mr. Nash proposed a vote of thanks to the Chairman, which Sir Arthur Spencer Wells seconded. Patients to the number of 542 have been treated in the hospital during the year. The duration of their stay was about four weeks, at an average cost of £10 apiece. The number of out-patients was 8,093. The out-patients' department has been overcrowded, and money is badly wanted to provide adequate accommodation; but in a year or two the authorities would be in a position to build if they had it. The Prince of Wales's Hospital Fund had made a donation of £500, after a long and minute investigation into the working of the hospital. This fact is a guarantee of the merits of the institution. It being a free hospital, and receiving patients from all parts of the kingdom, it appeals to the provinces for aid as well as the Metropolis. The income of the hospital from all sources, including legacies and sale of securities, reached £7,906, while the expenditure exceeded that amount by £88. Amongst the extraordinary expenditure must be noted the purchase of securities to the amount of £1,000, and the repayment of the debt to the bankers of £700. The hospital has an exceedingly comfortable and attractive appearance, and the patients seemed cheerful and happy.

